# Ohmic-Rectifying Conversion of Ni Contacts on ZnO and the Possible Determination of ZnO Thin Film Surface Polarity

**DOI:** 10.1371/journal.pone.0086544

**Published:** 2014-01-23

**Authors:** Kim Guan Saw, Sau Siong Tneh, Gaik Leng Tan, Fong Kwong Yam, Sha Shiong Ng, Zainuriah Hassan

**Affiliations:** Nano-optoelectronics Research Laboratory, Universiti Sains Malaysia, Minden, Penang, Malaysia; Gazi University, Turkey

## Abstract

The current-voltage characteristics of Ni contacts with the surfaces of ZnO thin films as well as single crystal (0001) ZnO substrate are investigated. The ZnO thin film shows a conversion from Ohmic to rectifying behavior when annealed at 800°C. Similar findings are also found on the Zn-polar surface of (0001) ZnO. The O-polar surface, however, only shows Ohmic behavior before and after annealing. The rectifying behavior observed on the Zn-polar and ZnO thin film surfaces is associated with the formation of nickel zinc oxide (Ni_1-x_Zn_x_O, where x = 0.1, 0.2). The current-voltage characteristics suggest that a p-n junction is formed by Ni_1-x_Zn_x_O (which is believed to be p-type) and ZnO (which is intrinsically n-type). The rectifying behavior for the ZnO thin film as a result of annealing suggests that its surface is Zn-terminated. Current-voltage measurements could possibly be used to determine the surface polarity of ZnO thin films.

## Introduction

In recent years, development in optoelectronic devices using ZnO has attracted great interest as this semiconducting material has a wide bandgap (∼3.4 eV), a high exciton binding energy (∼60 meV) and a relatively non-toxic nature [1]. ZnO normally crystallizes into the wurtzite structure but the ratio of its lattice parameters *c*:*a* is 1.602, which is lower than that for perfectly hexagonally close-packed atoms (1.633). The smaller ratio of the lattice parameters and the ionic nature of the Zn-O bond as well as the lack of inversion symmetry result in a net dipole moment along the *c*-axis of the unit cell. While the dipole moments cancel each other in the bulk they cause equal and opposite bound polarization charges on the Zn-polar and O-polar surfaces. The surfaces of single crystal (0001) ZnO can exist as Zn- or O-terminated. A previous study on the surface of ZnO thin film identified it as O-terminated [2]. Sasaki *et al.* [3], however, reported that the polarity of ZnO thin films depends on the growth temperatures. The electrical behavior of metal contacts on the different surfaces of ZnO may exhibit Ohmic, near-linear or rectifying behavior. For instance, Pt contact on ZnO thin film exhibits a rectifying behavior while Al turns out to be Ohmic in nature [4]. Intrinsic ZnO typically exhibits n-type conductivity which is attributed to donor defects such as interstitial Zn, oxygen vacancy or hydrogen [5]–[8]. Thus undoped ZnO in the form of single crystals or thin films is typically n-type. In many cases the successful application of ZnO-based devices would depend on the understanding and control of the current-voltage (I–V) characteristics of the metal films that act as electrical contacts and the related surface physics and chemistry involved during the contact formation. The Schottky model suggests that when a metal is in contact with a semiconductor, the Ohmic or rectifying character of the contact is dependent on the work function of the metal and the electron affinity of the semiconductor. A metal-semiconductor junction is Ohmic if the barrier formed by the contact is zero. In n-type semiconductors, an Ohmic junction happens when the work function of the metal is close to or smaller than the electron affinity of the semiconductor. It is important that in the fabrication of semiconductor devices, care is taken to ensure that Ohmic contacts are not inadvertently created where a rectifying connection is desired and vice versa. A recent study showed a conversion from Ohmic to rectifying behavior can occur as a result of remote room temperature O_2_/He plasma treatment [9]. Recent studies have largely focused on multilayer or bilayer metallization schemes involving pre- and post- deposition treatments which resulted in different I–V behaviors [10]–[12]. There were also some studies on single metals such as Cu, Ag, Au, Pd and Pt on either the thin film surface or on the polar surfaces of single crystal ZnO [13]–[18]. An important transition metal that is widely used as contact material is Ni. However, investigation into the use of this transition metal remains inconclusive. Ni/Au bilayer contacts on ZnO have yielded different results upon annealing. Motayed et al. [19] reported that as-deposited Ni/Au contacts and those annealed below 800°C demonstrated a near-linear behavior. A conversion to linear behavior was observed for 800°C while a higher annealing temperature started to degrade electrical properties as well as the surface morphology of the contacts. Ryu et al. [20], however, noted that the Ni/Au contacts remained linear before and after annealing at high temperatures although a lower contact resistance is associated with annealing at 600°C. It should be noted that the possibility of Ni contacts being oxidized to NiO (stoichoimetric or non-stoichoimetric oxides) could be partly the reason for the different I–V behaviors. Initially regarded as a Mott insulator, NiO has also been reported to be semiconducting in certain conditions [21]–[22]. For instance, a p-NiO/n-ZnO diode was fabricated recently by Ohta et al. [22]. Charge transport in NiO that shows p-type conductivity is attributed to thermally-activated hopping. NiO has a direct band gap of 3.7–4.0 eV with a cubic lattice.

Successful applications of ZnO devices with metal contacts such as Ni would therefore depend on a deeper understanding on the possible changes in the I–V characteristics of the contacts that can be caused by thermal annealing, annealing ambient, metallization schemes, defects as well as surface properties. Even low temperature post annealing of metal contacts on ZnO at 200–300°C has been known to cause a deterioration in the rectifying properties of metal contacts deposited on ZnO [23]. It should be noted that in a recent review on Ohmic and Schottky contacts on ZnO, work on Ni single metallization scheme on ZnO has been remarkably rare [11]. It would be interesting to know whether thermal annealing will change the metallic nature of the Ni contacts and induce the formation of a compound that would trigger a change in the I–V characteristics.

This work intends to investigate the I–V characteristics of Ni contacts on the surface of ZnO thin films and single crystal (0001) ZnO as well as changes in the electrical and accompanying structural properties as a result of thermal annealing. The Ni metallic layer is evaporated on the ZnO thin film (that is supported by sapphire substrate) as well as on both the Zn- and O-polar surfaces of the single crystal (0001) ZnO. The unit cell of Ni is a face-centered cube with the lattice parameter of 0.352 nm while ZnO has a hexagonal structure with lattice parameters *a* = 3.249Å and *c* = 5.206Å. Ni thin films have been known to oxidize to NiO as a result of annealing at low temperatures of less than 400°C [24] while ZnO has been known to decompose at temperatures lower than those allowed by thermodynamic data with evidence of Zn and O outdiffusion [25]. In addition, comparison of the I–V behavior and the accompanying material changes with those obtained from the Zn- and O-polar surfaces may provide a relatively simple method of indicating the surface polarity of ZnO thin films. So far, two known methods of determining the polarity of ZnO involve co-axial impact-collision ion scattering spectroscopy (CAICISS) [2]–[3] and etching with acid solutions [26]–[28]. The former is a sophisticated technique that uses the interaction of an ion beam and the surface atoms of the ZnO thin film in an ultrahigh vacuum chamber. In the latter method, Endo et al. [28] reported that the surface polarity of (0001) single crystal ZnO can be identified by looking at the pit density after etching it in an acid solution since the O-polar surface has a higher density of etch pits. Since the depth of a typical pit could be more than 1 µm it is impossible to use the etch method to determine the polarity of ZnO thin films. In addition, ZnO thin films have grain boundaries and defects that will affect the etching rate and surface characteristics making the task of distinguishing craters from hillocks unreliable. It should be noted that the polarity of ZnO thin films can be elusive. Previously it was widely believed that the polarity of ZnO thin film is – c (O-plane terminated) but recently Sasaki et al. [3] reported that ZnO thin films deposited directly on sapphire (0001) substrate at room temperature demonstrate a mixture of +c (Zn-plane termination) and –c (O-plane termination) polarity while films deposited on NiO buffer layer exhibit +c polarity. However, ZnO films deposited at high temperatures (∼600°C) are found to have – c polarity regardless of whether there is a NiO buffer layer or not. These findings imply that Ni contacts on the different types of surface polarity may exhibit different I–V behaviors. The outdiffusion of Zn and O from the different types of surface polarity may affect the Ni contacts differently, resulting in a distinct I–V characteristic.

## Materials and Methods

I–V characteristics were investigated by evaporating metallic Ni on sputtered ZnO thin films supported by α-(0001) sapphire. A mask was used to create areas or regions of metallic Ni on ZnO. The thickness of the ZnO film is ∼200 nm. A co-planar configuration was used in this work. I–V measurements were taken before and after the samples were annealed in a controlled furnace at 800°C for 2 h in a nitrogen atmosphere. The flow rate of the pure nitrogen gas was 4 L/min. All I–V measurements were taken at room temperature. The x-ray diffraction (XRD) 2θ measurements were taken with the X’Pert PRO system. The XRD data were collected using the Cu Kα radiation with a 0.02^o^ 2θ step size, and a 3 s count time. The annealing temperature was chosen after considering the XRD data which indicated that as-deposited ZnO thin films annealed at 800°C experienced a decrease of ∼1.4% in the *c* lattice constant to values comparable to the strain-free lattice constant (∼0.5205 nm). The higher *c* lattice constants of the as-deposited films indicated that the ZnO unit cell was elongated along the direction of growth and implied that the films were compressively strained. The annealing temperature for ZnO thin films was thus maintained at 800°C. The annealing temperature of 800°C is also chosen for the following reasons:

- ZnO is known to suffer an onset of decomposition reaction and surface degradation beginning with 900°C with preferential oxygen loss and visible metallic Zn droplets or islands forming on the surface as evident from x-ray fluorescence [23]. Therefore annealing at 900°C or higher is avoided for this work.
- The diffusion of Ni in a thin film appears to be significant at the annealing temperature of 800°C in an inert atmosphere. XPS analysis shows a mixture of Ni^0^ and Ni^2+^ species. No diffusion of Ni is observed at a lower temperature of 600°C. Annealing at this temperature is thus interesting as there are several possibilities involving Ni, interstitial Zn and O [29]. Annealing below 600°C is also avoided for this work as it will not involve any diffusion of Ni, which is our contact metal to be investigated in this work.
- A linear-nonlinear change in the I–V characteristics occurs for Ni/Au contacts on GaN at 800°C with evidence of reaction of Ni and Ga. No changes are found for lower annealing temperatures of 600 and 700°C [19]. A higher annealing temperature of 850°C degrades the linear characteristics into non-linear again. A similar phenomenon for Ni and ZnO is likely to occur.
- Annealing at 800°C is therefore likely to see the effect of Ni movement as well as the outdiffusion of both Zn and O atoms toward the metal layer.

Similar depositions of Ni were also done on (0001) single crystal ZnO measuring 5 mm×10 mm×0.5 mm that had been cleaned ultrasonically using solvents and blown dry using nitrogen gas for comparison. High quality single crystal (0001) ZnO that was hydrothermally grown where both the Zn and O-polar surfaces were epi-polished was used in this work. The root-mean-square (rms) roughness of the surface as measured by atomic force microscopy (AFM) was typically less than 2 Å. No pits were observed on the as-received Zn and O-polar surfaces. In addition no metallic contamination was found by energy dispersive spectroscopy (EDS) on both these surfaces. The Zn-polar and the O-polar surfaces could be easily identified by the direction of a beveled edge on the (0001) ZnO wafer. They can also be identified by observing the surface morphology after HCl etching. The morphology of the Zn-polar surface is characterized by isolated hexagonal pits of various sizes while the O-polar surface is heavily pitted after being etched in 0.22 M HCl for 1 min at 60°C. The depth of a typical etch pit is more than 1 µm.

The x-ray photoelectron spectroscopy (XPS) measurements were taken using a monochromatised Al Kα x-ray (hν = 1486.6 eV) source. The XPS spectrometer (Kratos AXIS Ultra Imaging Instrument) which was equipped with a hemispherical analyzer was operated at 150 W. The area of analysis was 110 µm×110 µm. Charge compensation was done using a flood gun. The single element scans were performed at constant pass energy of 20 eV. Charge correction was done using the C 1s position of adventitious carbon at the binding energy of 284.5 eV. All the analyses were done at room temperature.

## Results and Discussion

**Figure 1:**
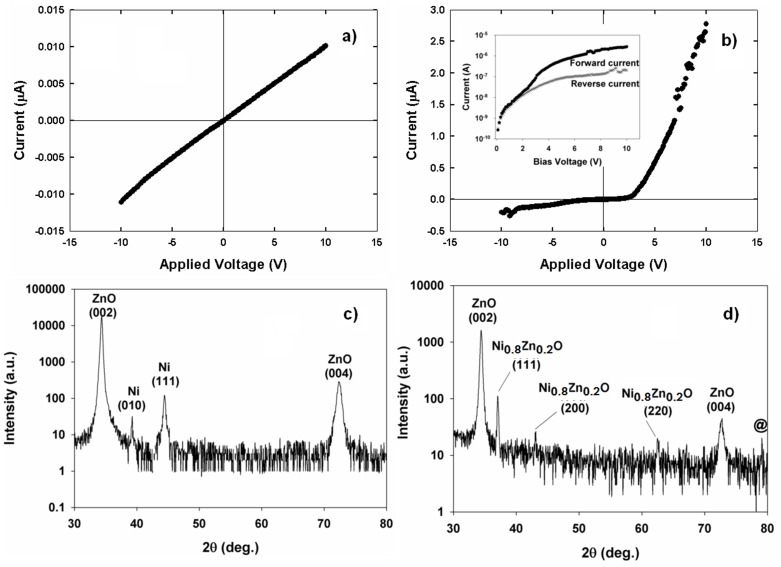
(a) I–V characteristics of Ni contacts on ZnO thin film before annealing. (b) I–V characteristics after annealing at 800°C. (c) XRD 2-theta pattern before annealing. (d) XRD 2-theta pattern after annealing at 800°C.

Fig. 1(a) shows the Ohmic behavior of evaporated Ni contacts on the ZnO thin film before annealing while Fig. 1(b) shows the forward and reverse I–V characteristics after annealing. The annealing process results in the conversion of the Ohmic behavior to a rectifying feature. The threshold voltage, V_TH_, is 3.50 V while the corresponding current is 1.71×10^−7^A. The sputtered ZnO thin film shows a preferential c-axis orientation. In principle, an Ohmic contact is formed when the work function of the evaporated Ni is less than the electron affinity of ZnO. Ni is known to have work functions in the range of 4.1–5.4 eV [30]. Evaporated Ni thin films are believed to have work functions less than 4.5 eV due to the atomically rough surface while the electron affinity of ZnO is around 4.5 eV [31].

The hexagonal Ni (010) and cubic Ni (111) phases of metallic Ni at 2θ = 39.262^o^ and 44.438^o^ as well as the wurtzite phase ZnO (002) and (004) peaks belonging to the ZnO thin film before annealing can be identified in the XRD pattern in Fig. 1(c). The XRD phase analysis in Fig. 1(d) shows that the metallic Ni phases have disappeared after annealing. However, both the ZnO (002) and (004) peaks are still present. The XRD analysis suggests that the conversion of Ohmic to a rectifying behavior is associated with the transformation of Ni into a nickel zinc oxide (Ni_x_Zn_1-x_O) compound with a cubic crystal system. The diffraction peaks observed at 2θ of 37.052^o^, 43.060^o^, 62.533^o^, and 79.075^o^ (labeled as @) can be indexed to the (111), (200), (220) and (222) phases of Ni_0.8_Zn_0.2_O, which belongs to the Fm–3m space group with lattice contacts *a* = *c* = 0.42010 nm (JCPDS ref no. 01-075-0271). The source of oxygen for the formation of Ni_0.8_Zn_0.2_O is believed to come from residual oxygen in the furnace while the source of Zn comes from the thermal diffusion of Zn. The movement of Zn is a possible phenomenon since the migration energy of Zn interstitials is only 77 kJ/mol [32]. The oxidation of Ni follows equation (1).
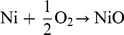
(1)


The oxidation of the Ni contact is mainly attributed to residual oxygen in the furnace. There is evidence for the possibility of oxidation by residual oxygen since it is found that a separate sample of Ni thin film on (0001) sapphire substrate is oxidized to NiO under similar conditions. A single XRD peak attributed to NiO (111) is observed at 2θ = 37.296^o^. Under normal conditions Ni forms only a single oxide, which is NiO. In the case of the oxidation of Ni to Ni_0.8_Zn_0.2_O, some of the oxygen that is involved in the oxidation process could also have originated from the decomposition of ZnO. The presence of Zn in Ni_0.8_Zn_0.2_O is believed to have come from the outdiffusion of Zn. It is well-known that ZnO does not sublime (equation (2)) but decomposes into gaseous products of oxygen and zinc vapour when heated at temperatures around 1700°C [33] according to equation (3):
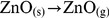
(2)

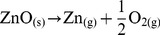
(3)


However, it should be noted that there is evidence from previous studies that outdiffusion of Zn as well as O (including O evolvement) begins at a much lower temperature [11]–[12], [25], [34]. Auger depth profiling in a recent study provides evidence of ZnO dissociation with O outdiffusion starting at 300°C [11]–[12] while secondary ion mass spectroscopy (SIMS) measurements indicate the outdiffusion of Zn and O for ZnO samples annealed at 700°C [34].

**Figure 2:**
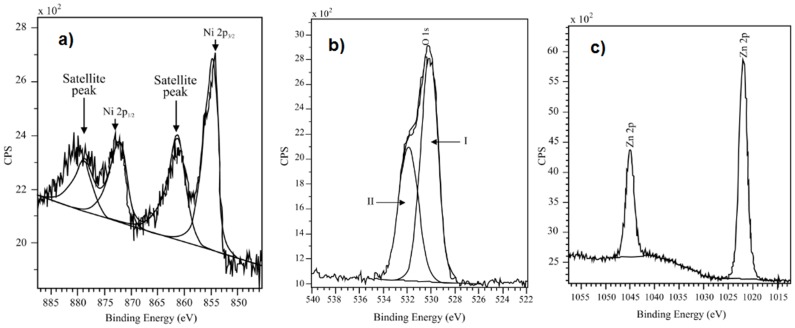
(a) XPS Ni 2p regional scan. (b) O 1s regional scan. (c) Zn 2p regional scan.

The two-valence oxidation state of Ni is confirmed by XPS measurements with the Ni 2p_3/2_ and Ni 2p_1/2_ peaks appearing at the binding energies of 854.0 and 872.5 eV, respectively. The intense Ni 2p_3/2_ single peak at 854.0 eV instead of 852.3 eV, which is the metallic state, suggests that the metallic Ni contact is oxidized to Ni^2+^ after the annealing process [35]–[36]. The binding energy positions of the Ni 2p_3/2_ and Ni 2p_1/2_ peaks are different from those observed for Ni and Ni_2_O_3_, confirming that Ni is present in a chemical state of 2^+^ and not as 3^+^ or 0. Two additional satellite peaks due to the shake-up process are found at 861.5 and 879.0 eV. The shake-up process peak is common in transition metal oxide materials. It is possible that the high temperature annealing induces a significant diffusion of Zn resulting in the replacement of Ni by Zn in the NiO cubic lattice. The resultant material, Ni_0.8_Zn_0.2_O, which also has a cubic structure suggests that NiO is first formed and acts as the host lattice with the subsequent incorporation of Zn atoms. It should be noted that the lattice constant of Ni_0.8_Zn_0.2_O (*a* = 0.42010 nm) is slightly larger than that of NiO (*a* = 0.41771 nm). This interesting phenomenon of lattice enlargement is also observed in the Ni/GaN system where the parameter of the Ni host lattice increases to accommodate the increased solubility of Ga in Ni as a result of high temperature annealing [37]. The XPS O 1s asymmetric peak can be deconvoluted into two components centered at 530.0 (component I) and 532.0 eV (component II), respectively. The former originates from Ni_0.8_Zn_0.2_O while the latter is attributed to chemisorbed oxygen, possibly carbonate-like as well as surface hydroxyl species from the ambient. Since the O 1s peak from an *in-situ* cleaved NiO is centered at 529.4 eV [38], we suggest that the incorporation of Zn into the NiO lattice has shifted the O 1s peak slightly towards higher binding energy. Evidence from the core scan of the Zn 2p region collaborates with the information from the O 1s spectrum. The Zn 2p spectrum consists of one set of doublets where the Zn 2p_3/2_ and Zn 2p_1/2_ are positioned at 1021.0 and 1044.5 eV, respectively. It should be noted that the position of the Zn 2p_3/2_ peak is shifted from that of Zn in the elemental as well as oxide form typically observed at 1021.4–1021.7 eV [39]. The shift to a lower binding energy of 0.7 eV collaborates with the XRD evidence of the formation of a new nickel zinc oxide compound. Fig. 2(a) – (c) show the XPS spectra.

The Ohmic-rectifying conversion is an interesting phenomenon as annealing is sometimes thought to result in a linear I–V characteristic due to the diffusion of metal from the metal contacts. However, the interpretation that metal diffusion makes the contacts exhibit Ohmic behavior may not apply in our present work for the following reasons:

- Previous studies that attribute linear behavior after annealing to the diffusion of metal are based on bilayer metal contacts such as Ni/Au and Ti/Au [12], [19]. Motayed et al. [19] observed that non-annealed Ni/Au contacts and those annealed at 600°C and 700°C were non-linear but became Ohmic after annealing at 800°C and attributed this phenomenon to the diffusion of Ni and subsequent interfacial reaction involving Ni. It should be noted that not all bilayer metal contacts exhibit nonlinear-linear conversion as a result of high temperature annealing. For instance, Ryu et al. [20] found that non-annealed Ni/Au contacts are already Ohmic and remain so after annealing at 500°C and 600°C.

Bilayers usually use Au or Pt and these layers are used to prevent surface oxidation. However, in our present work, since we do not use Au or Pt to prevent oxidation, our Ni thin film is capable of being completely oxidized, resulting in a I–V behavior that is different from the bilayers.

- Previous studies that observed a change from non-linear to Ohmic behavior after annealing involved a very short period of annealing of several seconds to about 1 min. Our present work involves a much longer period of annealing of 2 h. In particular, the longer period of annealing together with a higher temperature is sufficient for the complete transformation of the Ni thin film as evident in the XRD analysis.

In this work, the rectifying I–V behaviour is attributed to the formation of a p-n junction involving Ni_0.8_Zn_0.2_O as a p-type material and ZnO that is intrinsically n-type. The assumption that Ni_0.8_Zn_0.2_O is a p-type semiconductor is plausible since NiO is sometimes observed to exhibit p-type conducting properties although it is generally regarded as a Mott insulator [22]. The migration of and incorporation of Zn into the host lattice of NiO that eventually results in the formation of nickel zinc oxide would certainly enhance the p-type semiconducting property. The non-abrupt threshold voltage also implies the presence of some defect states. Further insights can be obtained from similar experiments on the Zn- and O-polar surfaces of single (0001) crystal ZnO.

**Figure 3:**
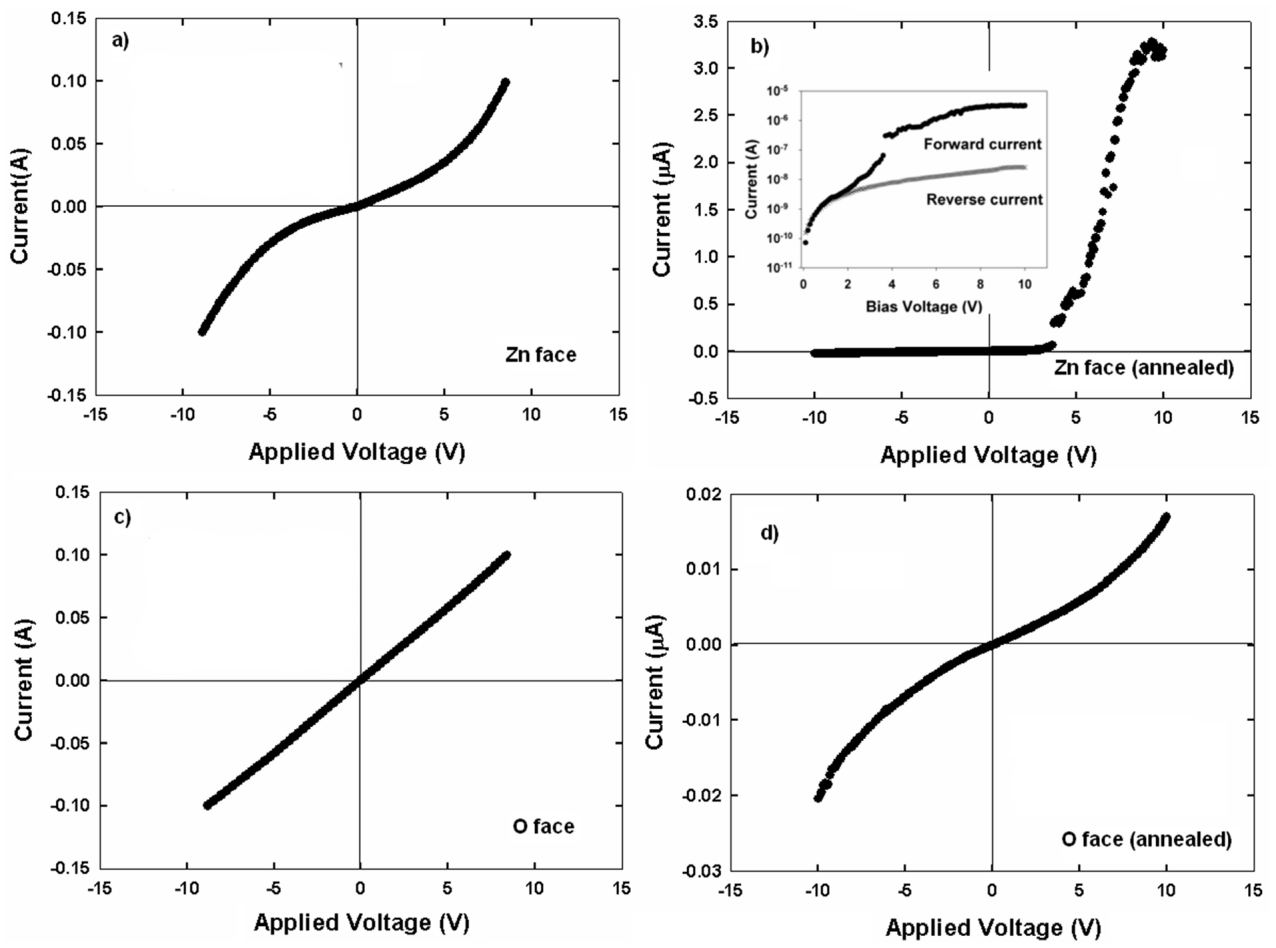
I–V characteristics of the Ni contact on (a) Zn-polar surface before annealing; (b) Zn-polar surface after annealing; (c) O-polar surface before annealing; (d) O-polar surface after annealing.

**Figure 4:**
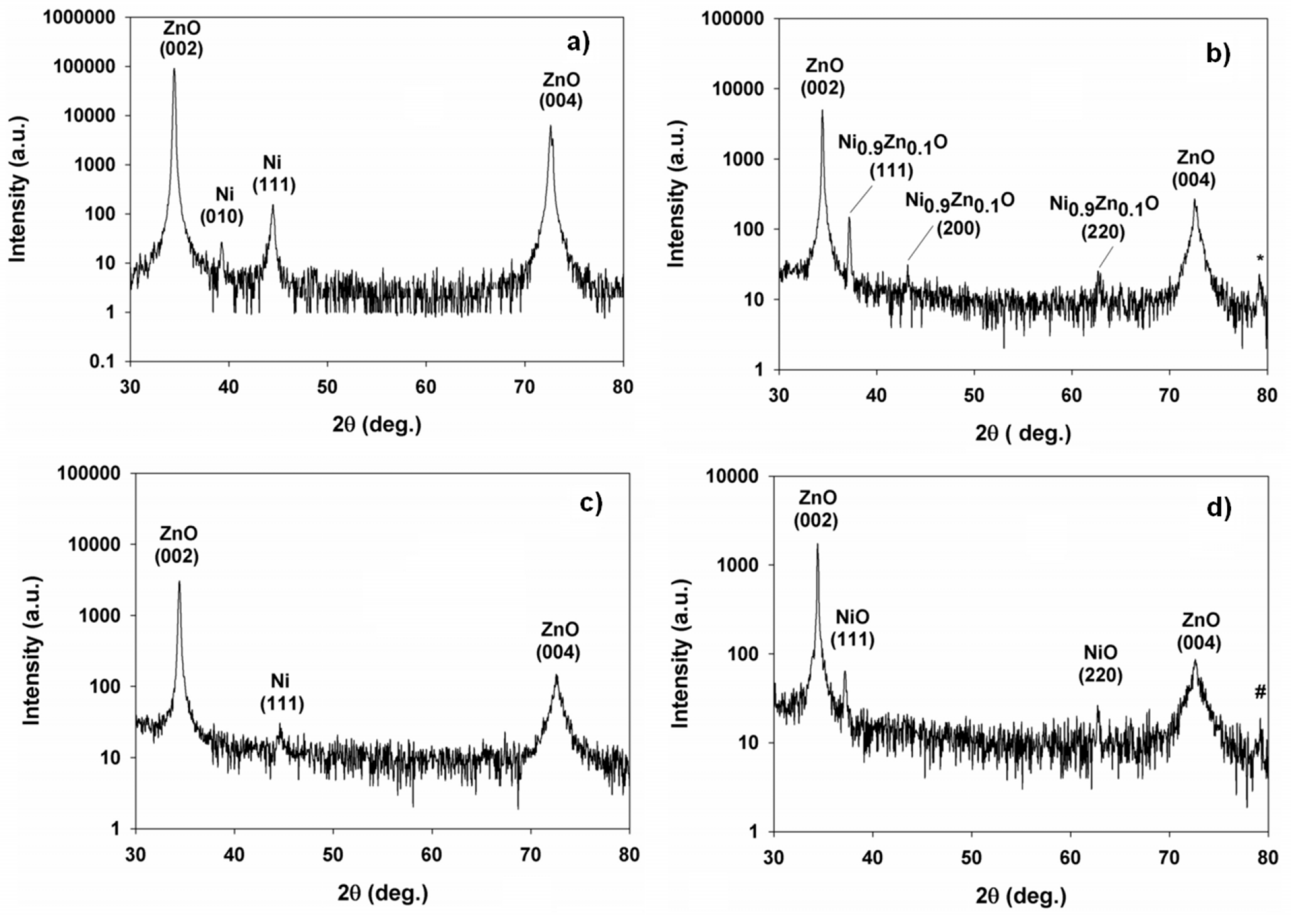
XRD patterns of the Ni contact on (a) Zn-polar surface before annealing; (b) Zn-polar surface after annealing; (c) O-polar surface before annealing; (d) O-polar surface after annealing.

Fig. 3 (a) and (b) show the I–V characteristics of the Ni contacts on the Zn-polar surface of (0001) single crystal ZnO before and after annealing, respectively. An Ohmic I–V behavior is observed for the as-received Zn-polar surface. Interestingly, an Ohmic-rectifying conversion similar to that for ZnO thin films is observed for the Zn-polar surface as a result of annealing. The threshold voltage is 3.75 V. Fig. 3 (c) and (d) refer to I–V characteristics of the O-polar surface of (0001) single crystal ZnO before and after annealing, respectively. The as-received O-polar surface shows an Ohmic behavior but no Ohmic-rectifying conversion is observed as a result of annealing. XRD patterns in Fig. 4 (c) and (d) reveal that the Ni contacts on the O-polar surface has been oxidized to NiO as a result of annealing. The diffraction peak at 2θ = 44.6445^o^ in Fig. 4(c) is attributed to (111) Ni (JCPDS ref no. 01-087-0712) while the peaks at 2θ = 37.207^o^, 62.806^o^ and 79.375^o^ (labeled as #) in Fig. 4(d) can be indexed to (111) NiO, (220) NiO and (222) NiO respectively (JCPDS ref no. 047-1049). These peaks indicate the face-centered cubic structure of NiO where the lattice parameter *a* is 0.41771 nm. Based on the XRD results the slight degradation of the linear behavior seen on the O-polar surface after annealing is probably caused by the oxidation of the Ni contacts to NiO. Without the incorporation of Zn, an Ohmic behavior is still observed for NiO, indicating that NiO is not totally insulating. The formation of NiO indicates that oxygen is outdiffused from the ZnO layer, resulting in the accumulation of oxygen vacancies near the ZnO surface. Oxygen vacancies were reported to act as donors in ZnO. Thus the increase in the carrier concentration near the surface of the ZnO layer could be responsible for the Ohmic behavior albeit with a high resistance. The XRD analysis of the Zn-polar surface (Fig. 4(a) and (b)), however, reveals that nickel zinc oxide, Ni_0.9_Zn_0.1_O, has been formed instead of NiO. Peaks corresponding to (111) Ni_0.9_Zn_0.1_O, (200) Ni_0.9_Zn_0.1_O, (220) Ni_0.9_Zn_0.1_O and (222) Ni_0.9_Zn_0.1_O (labelled as *) (JCPDS ref no. 01-075-0270) are found besides two prominent peaks belonging to (002) and (004) ZnO (Fig. 4(b)). The XRD analysis indicates that after annealing, nickel zinc oxide (Ni_1-x_Zn_x_O, where x = 0.1, 0.2) could only be observed in the ZnO thin film and the Zn-polar surface but not on the O-polar surface. By comparing the current values at +10V (Fig. 2(b), 3(b) and (d)) it is obvious that NiO is more resistive than Ni_0.9_Zn_0.1_O and Ni_0.8_Zn_0.2_O by a few orders of magnitude. This also implies that NiO behaves more like an insulator rather than a p-type semiconductor and therefore no rectifying I–V characteristics are observed. The inclusion of Zn into the host lattice of NiO, however, causes the I–V characteristics to change to a rectifying nature. Evidence from the I–V and XRD measurements suggests that in both cases Ni_0.9_Zn_0.1_O and Ni_0.8_Zn_0.2_O form a p-n heterojunction with ZnO. As p-type semiconductors, the difference in the bandgap between Ni_0.9_Zn_0.1_O and Ni_0.8_Zn_0.2_O can be determined from equations (4) and (5) which link the threshold voltage and the bandgap.
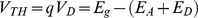
(4)

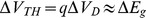
(5)where *V_TH_*, *V_D_* and *E_g_* refer to the threshold voltage, diffusion voltage and energy bandgap, respectively while 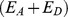 is assumed to be constant in Ni_0.9_Zn_0.1_O as well as Ni_0.8_Zn_0.2_O. From the threshold voltage values the difference in bandgap is 0.25 eV. Considering the difference in the bandgaps of NiO (∼4.0 eV) and ZnO (∼3.3 eV) is ∼0.7 eV, the obtained value of 0.25 eV for the difference in the bandgaps of Ni_0.9_Zn_0.1_O and Ni_0.8_Zn_0.2_O is expected. It also implies that Ni_0.9_Zn_0.1_O has a larger bandgap than Ni_0.8_Zn_0.2_O. A possible reason for the larger bandgap of the former is the inclusion of less Zn, which enables the material to possess a bandgap that is closer to that of NiO. Our work thus suggests that Ohmic-rectifying conversion of Ni contacts on ZnO is associated with the formation of nickel zinc oxide since its presence is detected in the ZnO thin film as well as the Zn-polar surface but not in the O-polar surface. It is highly likely that the surface of the ZnO thin film is Zn-terminated. While the use of I–V and XRD measurements at this stage seems to indicate a possible method to determine the surface polarity of ZnO either as a thin film or as a bulk single crystal, further investigation should be done with different annealing temperatures as well as with ZnO thin films that have O-polar surfaces. Further work should also be done to investigate whether this method could be used for other semiconductor materials.

## Conclusions

Ni contacts on ZnO demonstrate both Ohmic and rectifying behavior depending on the surface polarity of ZnO as well as thermal annealing. The as-sputtered ZnO thin film as well as the as-received Zn- and O-polar surfaces of (0001) ZnO generally show an Ohmic behavior. Ohmic-rectifying conversion is observed for the ZnO thin film and Zn-polar surface of (0001) ZnO that have been annealed and is associated with the formation of nickel zinc oxide (Ni_1-x_Zn_x_O, where x = 0.1, 0.2). No conversion is observed for the O-polar surface of (0001) ZnO. The Ni contacts are oxidised to NiO but no nickel zinc oxide has been detected. The surface of the sputtered ZnO thin film that has been annealed at 800°C is likely to be Zn-terminated.
